# Draft Genome Sequence of *Methanobrevibacter smithii* Isolate WWM1085, Obtained from a Human Stool Sample

**DOI:** 10.1128/genomeA.01055-17

**Published:** 2017-09-28

**Authors:** Matthew E. Jennings, Nicholas Chia, Lisa A. Boardman, William W. Metcalf

**Affiliations:** a Department of Microbiology, University of Illinois, Urbana, Illinois, USA; b Department of Surgery, Mayo Clinic, Rochester, Minnesota, USA; c Division of Gastroenterology and Hepatology, Mayo Clinic, Rochester, Minnesota, USA

## Abstract

*Methanobrevibacter smithii* is a common inhabitant of the human gut. Here, we present a draft genome sequence of *M. smithii* isolate WWM1085, obtained from a human stool sample. This sequence will improve our understanding of the genetic diversity of this human-associated methanogen.

## GENOME ANNOUNCEMENT

The human-associated methanogen *Methanobrevibacter smithii* is found in roughly 95% of individuals (1). This microorganism plays an important role in the gut ecosystem by consuming hydrogen produced by fermentative members of the intestinal microflora, preventing the stalling of metabolic processes due to high product concentrations (2). *M. smithii* obtains energy by reducing CO_2_ with electrons from hydrogen; however, other methanogens are known to utilize other substrates found in the human gut, such as methanol or methylamines (3–5). To gain insight into the diversity of human-associated methanogens, we isolated *Methanobrevibacter smithii* WWM1085 from a human stool sample (Mayo Clinic, biome number 101159) in the presence of CO_2_-H_2_ as a carbon and energy source. The isolate possessed autofluorescence at 420 nm, indicating the presence of the methanogen cofactor F_420_ (6).

Genomic DNA was isolated from pure culture using chloroform extraction following digestion with pseudomurine endoisopeptidase to digest the cell wall (7). The University of Illinois Core Sequencing Facility generated a genomic library using a Hyper prep kit (Kapa Biosystems, Wilmington, MA). The library was sequenced using Illumina MiSeq version 3 with 250-bp paired-end reads, resulting in 772,864 reads. Reads were trimmed using the BBDuk open-source software from the Joint Genome Institute (JGI), producing 564,368 usable reads. The reads were assembled using Geneious (version 9.1.4) (8), generating 132 scaffolds. Only scaffolds with coverage greater than 55-fold (mean coverage, 61.4-fold) were analyzed further, resulting in 16 scaffolds totaling 1.93 Mbp. Scaffolds were annotated using Rapid Annotations using Subsystems Technology (RAST), which identified 1,816 protein-coding genes, 10 rRNA genes, and 35 tRNA genes (9). Based on similarity to the 16S rRNA sequence from *M. smithii* ATCC 35061 (99%), the isolate was determined to be *M. smithii*. The assembled genome is slightly larger than the reference genome (*M. smithii* ATCC 35061), which is 1.89 Mbp and contains 1,667 protein-coding genes (3). Scaffolds were deposited in GenBank under accession number NQLD00000000.

Comparisons between the WWM1085 isolate genome and that of *M. smithii* ATCC 35061 revealed 153 genes unique to WWM1085 and 127 genes unique to ATCC 35061. The majority of those genes (72.5% and 68.5% for WWM1085 and ATCC 35061, respectively) are annotated as coding for hypothetical proteins. Differences between the two strains include the presence of unique restriction systems and insertion elements, as well as the presence of nine additional clustered regularly interspaced short palindromic repeat (CRISPR)-associated genes in WWM1085, which are absent in ATCC 35061 (the strains share six CRISPR-associated genes). This suggests a difference in exposure to foreign DNA elements between the two strains. Core methanogenesis genes are present in both genomes.

The isolate can grow using CO_2_-H_2_ as a carbon and energy source, similar to the parent strain. The isolate can grow in media without both yeast extract and tryptone. Additionally, no unique methyltransferase genes, which are required for growth on methylated compounds, were identified.

This additional genome increases our understanding of the genetic diversity of *M. smithii* within the human population. This knowledge will enable us to better understand the role of *M. smithii* in the human intestinal ecosystem and how that role may vary between individuals.

### Accession number(s).

This whole-genome shotgun assembly has been deposited in GenBank under accession number NQLD00000000. The version described in this paper is the first version.
